# Construction and validation of a three-dimensional CT spatial localization algorithm for segond fracture based on tibial bony landmarks: an anatomical reference study for AI-assisted preoperative planning

**DOI:** 10.3389/fmed.2026.1958990

**Published:** 2026-10-07

**Authors:** Ziteng Guo, Chenggang Yang, Yongchen Zheng, Qing Zhao, Ning Chen, Yan Ding, Qi Chen, Liwei Pan, Chen Zhou

**Affiliations:** 1 Department of Orthopedic Surgery II, Beijing Shunyi Hospital, Beijing, China; 2 School of Medical Technology, Capital Medical University, Beijing, China

**Keywords:** 3D CT analysis, AI surgical planning, bony anatomical reference, computational anatomy, digital twin, geometric inference model, segond fracture

## Abstract

**Background:**

The clinical translation of AI-assisted surgical navigation and digital twin modeling is hindered by the absence of a standardized, reusable anatomical spatial reference framework, as current one-dimensional or two-dimensional localization methods for the Segond fracture bed inadequately represent three-dimensional bony spatial relationships for intelligent navigation systems.

**Objective:**

To meet the needs of AI- and digital twin–driven precision orthopedics, this study constructs a CT-based three-dimensional coordinate system of the knee using tibial landmarks, applies a normalization algorithm for standardized Segond fracture localization, and validates a reusable anatomical reference model, thereby bridging the translational gap in AI localization and offering a standardized computational framework for knee surgical planning and digital twin modeling.

**Methods:**

A retrospective review was conducted on 30 patients with left-sided Segond fracture who underwent thin-slice knee CT examinations at Beijing Shunyi Hospital between June 2010 and December 2025. Patients were randomly assigned to a modeling group (*n* = 15) and an independent validation group (*n* = 15). Using 3D Slicer software, the following landmarks were annotated: the most lateral point of the fibular head (P1), the midpoint of the lateral edge of the tibial tubercle (P2), the most superior point of Gerdy's tubercle (P3), and the center of the Segond fracture bed (P4). A right-handed orthogonal local coordinate system was constructed with P2 as the origin, based on P1, P2, and P3, and scale normalization was performed using the distance from P2 to P1. The modeling group was used to estimate the preliminary single center anatomical prior of the fracture bed in the normalized tibial local coordinate system(N-TLS). In the validation group, only the native global RAS coordinates of P1, P2, and P3 were input, and the predicted native global RAS coordinates of P4 were obtained via inverse geometric mapping. Localization accuracy was evaluated using the three-dimensional Euclidean distance between the predicted coordinates and the CT-measured reference coordinates.

**Results:**

The mean normalized coordinates of the Segond fracture bed center in the modeling group were (0.8209, 0.4300, −0.0078). Based on this anatomical prior, the deterministic geometric estimator achieved a mean localization error of 3.21 mm in the 15-case single-center independent validation cohort, with a standard deviation of 1.23 mm, a median error of 2.75 mm, a maximum error of 5.33 mm, and a minimum error of 1.68 mm. These findings indicate that the Segond fracture bed center exhibits a population-level distribution pattern within this local coordinate framework that is amenable to geometric localization.

**Conclusion:**

This study established a novel three-dimensional CT-based computational coordinate system for the knee joint using tibial bony landmarks, characterized the standardized three-dimensional spatial distribution pattern of the center of the Segond fracture bed, and developed a geometric prediction algorithm model for the fracture center point. The model demonstrated favorable millimeter-level localization accuracy, providing a standardized computational anatomical reference for digital surgical planning and future knee digital twin modeling, and offering methodological support for evidence-based clinical decision-making and intelligent orthopedic healthcare systems.

## 1 Introduction

The deep integration of artificial intelligence and digital twin technologies is fundamentally reshaping the paradigm of precision orthopedic surgical planning (1). By constructing patient-specific digital twin models of the knee joint, surgeons can perform implant simulation fitting, ligament reconstruction tunnel planning, and biomechanical prediction in a virtual environment for patients with Segond fracture accompanied by high-grade pivot-shift test positivity (2, 3). Segond fracture is an avulsion fracture of the anterolateral tibial plateau and is commonly associated with injuries to the anterior cruciate ligament (ACL) and the anterolateral structures of the knee (4, 5). In clinical practice, for patients with Segond fracture presenting with severe pivot shift and high sports demands, concurrent anatomic reconstruction of the anterolateral ligament (ALL) is performed alongside ACL reconstruction (6, 7). Given that the fracture bed is adjacent to the tibial insertion of the anterolateral ligament (ALL), its spatial location serves both as an important imaging indicator for identifying anterolateral complex injuries of the knee and as a reference for fracture reduction and fixation as well as ALL anatomic reconstruction (8, 9). However, the implementation of AI-assisted preoperative planning for localizing Segond fracture faces a fundamental bottleneck: current localization methods for the Segond fracture bed in the knee joint rely on one-dimensional linear distance scalars or two-dimensional imaging-based approaches, which fail to fully characterize the three-dimensional spatial relationships of bony structures and cannot satisfy the requirement for unified input features in intelligent navigation systems. This limitation creates a significant translational gap among anatomical measurement, digital modeling, and intelligent planning. For computer-assisted surgical planning and AI-assisted navigation localization, there is a pressing need for a standardized three-dimensional localization framework that enables cross-patient comparison, supports inverse computation, and carries unambiguous anatomical meaning. To address the limitations of traditional measurement methods in adapting to AI and digital twin modeling, the present study selected three tibial bony landmarks with high identifiability and stable anatomical morphology to construct a local three-dimensional CT-based computational coordinate system. Through scale normalization, we quantified the preliminary single center anatomical prior spatial distribution of the Segond fracture bed center, established a deterministic deterministic geometric estimator based on preliminary single center anatomical prior, and evaluated its three-dimensional localization error in an independent validation cohort. This approach is expected to provide a standardized quantitative tool for AI-assisted preoperative planning and intraoperative minimally invasive navigation for Segond fracture, while also furnishing a unified anatomical computation module for the future development of digital twin platforms for knee injuries and AI-assisted preoperative planning.

## 2 Materials and methods

### 2.1 Patient selection criteria

This was a single-center, retrospective imaging study. Patients with Segond fracture who presented to the Department of Orthopedics at Shunyi Hospital, Beijing, between June 2010 and December 2025 and who had undergone thin-slice CT of the knee were consecutively retrieved from the institutional database. After screening, a total of 30 cases with left-sided Segond fracture were enrolled and subsequently allocated into a model-development group (*n* = 15) and an independent validation group (*n* = 15) according to a predefined randomization scheme.

In the model-development group, there were 8 males and 7 females, with a mean age of 36.5 ± 8.2 years (range: 22–58 years). The validation group comprised 7 males and 8 females, with a mean age of 35.9 ± 7.8 years (range: 23–57 years). No statistically significant differences were observed between the two groups with respect to sex or age (both *P* > 0.05).

The inclusion criteria were: (1) CT and MRI findings consistent with the diagnosis of left-sided Segond fracture; (2) CT slice thickness ≤1 mm, with image quality sufficient for three-dimensional reconstruction and reliable identification of osseous landmarks; and (3) intact anatomical integrity of the proximal tibia and fibular head. The exclusion criteria were: (1) concomitant comminuted or multiple fracture that altered the native morphology of the proximal tibia; (2) history of prior knee surgery; (3) presence of significant developmental deformity, advanced osteoarthritis, tumor, infection, or severe osteoporosis; and (4) motion or metallic artifacts that precluded accurate and reproducible identification of critical landmarks.

### 2.2 CT image acquisition and three-dimensional reconstruction

Imaging data were acquired using a Siemens spiral CT system. The CT scanner model was the Philips Incisive CT Power, with tube voltage of 80–140 kV, tube current-time product of 30–500 mAs (DoseRight automatic modulation), pitch of 0.5–1.5, reconstruction slice thickness of 0.625–1.0 mm (all ≤1.0 mm), and a matrix size of 512 × 512. The scan range extended from approximately 5 cm above the femoral condyles to approximately 5 cm below the tibial tubercle. Thin-slice DICOM data were imported into 3D Slicer 5.10.0 for multiplanar reconstruction and visualization of three-dimensional bone models. Bony landmarks were cross-checked on axial, coronal, sagittal planes and the 3D models. RAS global coordinates were exported with a consistent coordinate output method for all cases. The Markups point annotation module was used to localize tibial bony anatomical landmarks on 2D tomographic slices and reconstructed 3D bone models, enabling extraction of raw three-dimensional coordinates for all feature points.

### 2.3 Anatomical landmark identification and coordinate extraction

Three intraoperatively identifiable osseous anatomical reference points and one target point on the center of the Segond fracture bed were designated using triplanar (coronal, sagittal, and axial) CT views: P1, the most lateral point of the fibular head; P2, the midpoint of the most lateral margin of the tibial tuberosity; P3, the highest point of Gerdy's tubercle; and P4, the center of the Segond fracture bed (Figure 1). Thin-slice DICOM images from all participants were imported into 3D Slicer for three-dimensional (3D) reconstruction and manual annotation of the feature points. The native global RAS system coordinates of the four key points were extracted in a batch processing manner.

**Figure 1:**
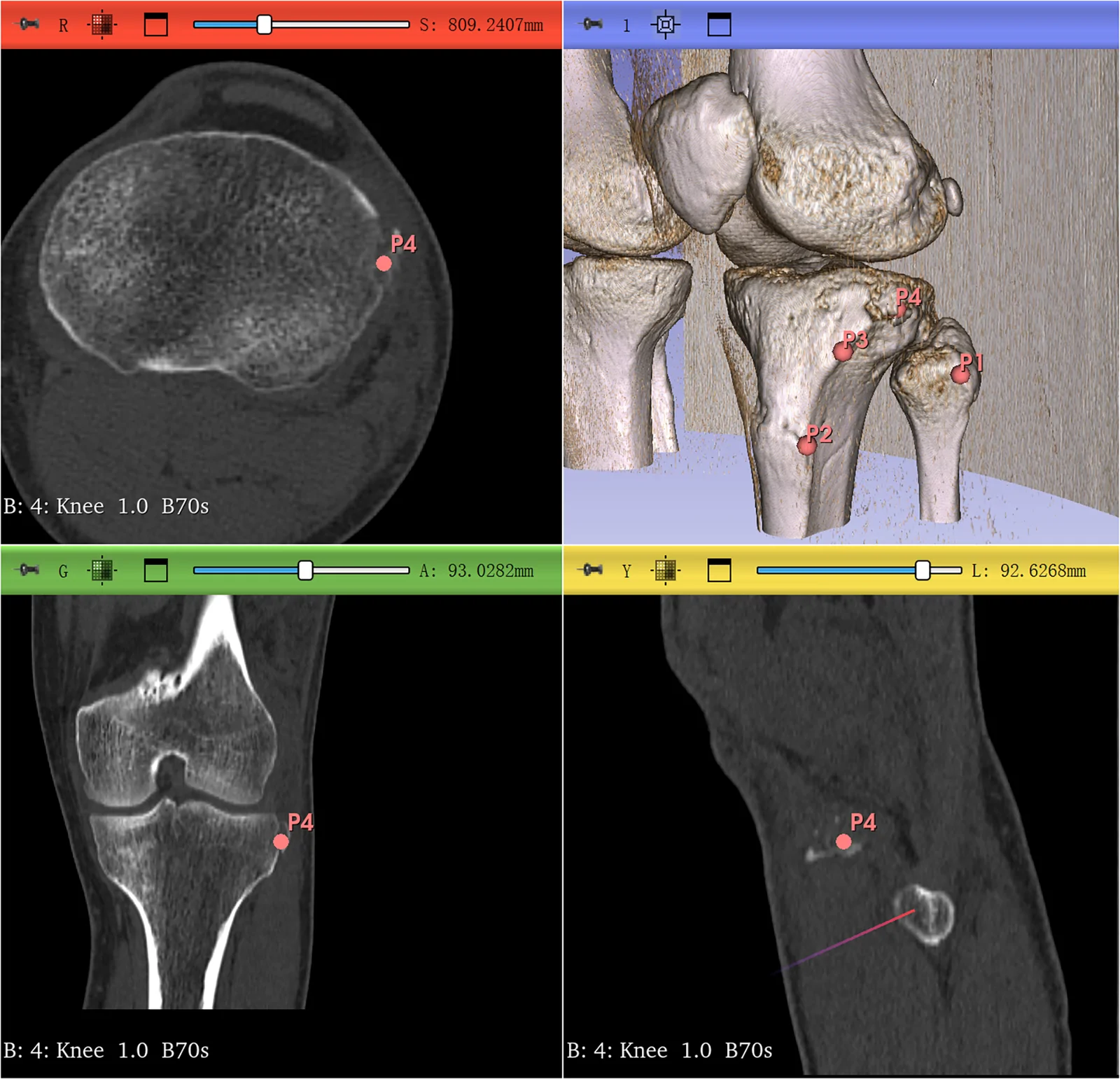
Annotation of P1–P4 on the three-dimensional knee model and localization of the segond fracture bed center (P4) on multi-planar CT images.

Three orthopedic surgeons proficient in knee-joint CT measurements performed manual landmark annotation of P1-P4 for 30 cases of Segond fracture involving the left knee. Each surgeon annotated all cases in triplicate. Intra-class correlation coefficients (ICCs) were calculated for the three-dimensional coordinates of each landmark. The resulting ICCs ranged from 0.99 to 1.00, indicating excellent intra-operator repeatability for landmark selection. The mean coordinate value from each surgeon's three repeated annotations was taken as that surgeon's final annotation result. The surgeon-specific mean landmark values from the three orthopedic surgeons were further arithmetically averaged to obtain the final reference coordinates of P1-P4 for each case.

### 2.4 Tibial local system construction

A three-dimensional computational framework for the tibial local system (TLS) was established based on the vector cross-product rules of spatial analytic geometry (Figure 2). The complete coordinate transformation logic was encoded into an automated computational program, providing a unified geometric transformation operator for AI model-based batch inference of knee anatomical landmarks.

**Figure 2:**
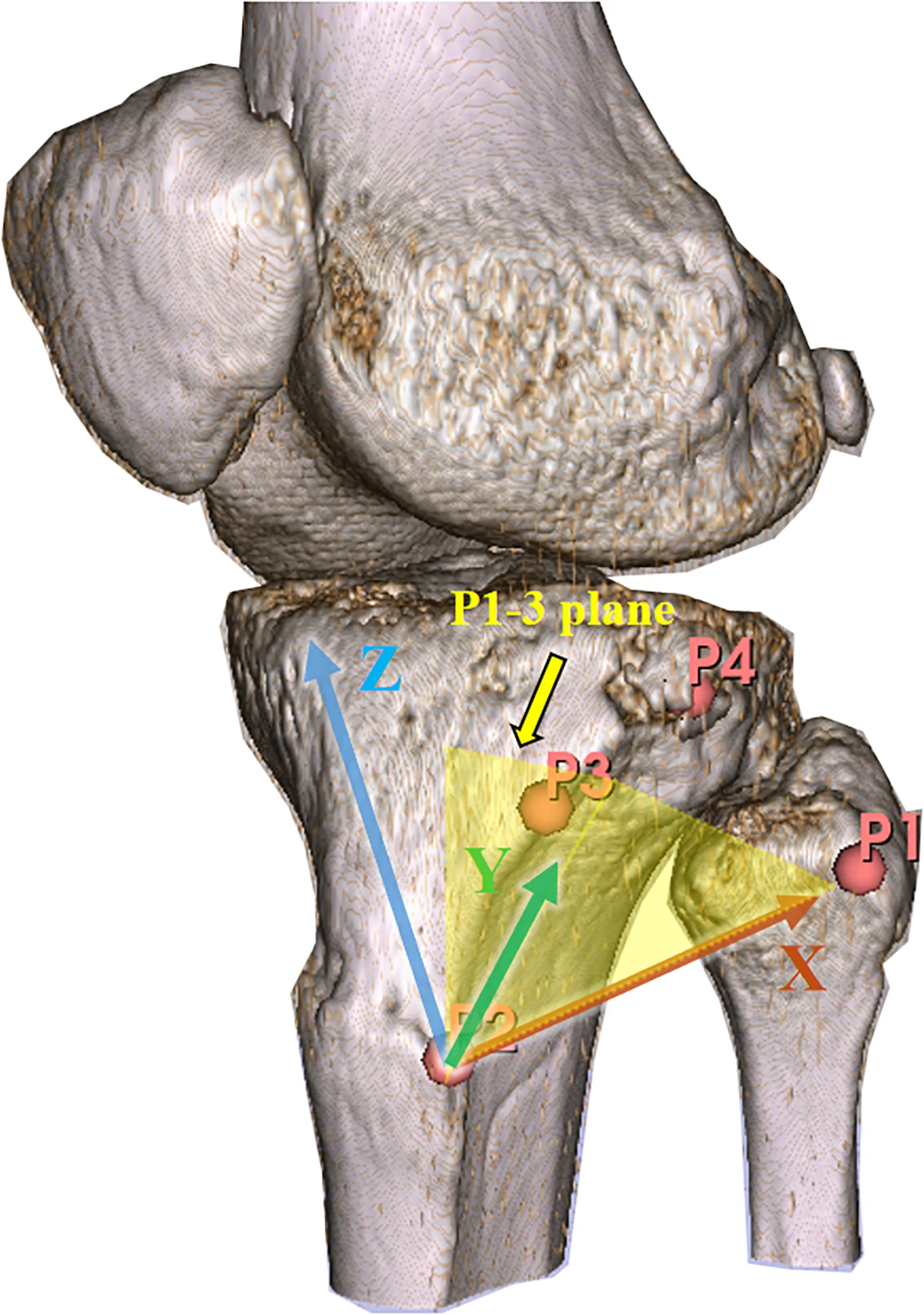
Establishment of the novel tibial local coordinate system in the three-dimensional knee model. P1, the most lateral point of the fibular head; P2, the midpoint of the most lateral margin of the tibial tuberosity; P3, the highest point of Gerdy's tubercle; P4, the center point of the Segond fracture bone bed. The plane (shaded) is defined by P1, P2, and P3.

#### 2.4.1 Establishment of the X-axis

The positive direction of the X-axis was defined as the direction from the tibial tuberosity (P2) to the fibular head (P1). The direction vector was calculated as $\vec{v_{x}}=P_{1}-P_{2}$, which was then normalized to obtain the unit vector $\vec{e_{x}}$ for the X-axis:

$$\vec{e_{x}}=\frac{\vec{v_{x}}}{\left|\vec{v_{x}}\right|}$$

#### 2.4.2 Establishment of the Z-axis

The Z-axis was defined as the direction perpendicular to the anatomical plane formed by the three points P1, P2, and P3, with its positive direction oriented toward the lateral aspect of the tibia. An auxiliary unit vector $\vec{v_{aux}}$ was first defined as the direction from the tibial tuberosity (P2) to Gerdy's tubercle (P3). The unit vector $\vec{e_{z}}$ for the Z-axis was then obtained by computing the cross product of the X-axis unit vector $\vec{e_{x}}$ and the auxiliary vector $\vec{v_{aux}}$, followed by normalization:

$$\vec{v_{aux}}=\frac{P_{3}-P_{2}}{\left|P_{3}-P_{2}\right|}$$

$$\vec{e_{z}}=\frac{\vec{e_{x}}\times \vec{v_{aux}}}{\left|\vec{e_{x}}\times \vec{v_{aux}}\right|}$$

#### 2.4.3 Establishment of the *Y*-axis

To ensure strict mutual orthogonality of the coordinate axes and compliance with the right-hand rule, the positive direction of the Y-axis was defined as pointing toward the posteromedial aspect of the tibia. The unit vector $\vec{e_{y}}$ for the Y-axis was obtained by computing the cross product of the Z-axis unit vector and the X-axis unit vector:

$$\vec{e_{y}}=\vec{e_{z}}\times \vec{e_{x}}$$

### 2.5 Tibial local system coordinate extraction and scale normalization

#### 2.5.1 Coordinate extraction of the center of the segond fracture bed in the tibial local system

Following the construction of the TLS, spatial feature extraction of the Segond fracture bed key point (P4) was performed. The displacement vector of the fracture fragment relative to the origin (the tibial tuberosity, P2) was first calculated as $\vec{v_{4}}=P_{4}-P_{2}$ Subsequently, via dot-product projection operations, the native global RAS system imaging space vector was mapped onto the TLS, thereby completing the geometric feature encoding. The local coordinate features of the fracture bed, denoted as $\left(a_{x},a_{y},a_{z}\right)$, were output as follows:

$$a_{x}=\vec{v_{4}}\cdot \vec{e_{x}}$$

$$a_{y}=\vec{v_{4}}\cdot \vec{e_{y}}$$

$$a_{z}=\vec{v_{4}}\cdot \vec{e_{z}}$$

#### 2.5.2 Normalization of the center of the segond fracture bed position

To eliminate feature bias arising from inter-individual variations in skeletal dimensions, the tibial anatomical reference length L (the reference distance from the tibial tuberosity to the lateral aspect of the fibular head) was selected as the scale normalization factor. The absolute local coordinates obtained from the encoding output were then scaled using L to yield the normalized tibial local system (N-TLS) coordinates $\left(a_{x}^{\prime },a_{y}^{\prime },a_{z}^{\prime }\right)$:

$$\$(a_{x}^{\prime },a_{y}^{\prime },a_{z}^{\prime })=\left(\frac{a_{x}}{L},\frac{a_{y}}{L},\frac{a_{z}}{L}\right)\$$$

### 2.6 Anatomical prior and deterministic geometric estimator

The arithmetic mean of the normalized coordinates from all cases in the model-development group was calculated to obtain the standard anatomical prior center of the Segond fracture bed in the tibial local coordinate system $\left({\bar{a}}_{x}^{\prime },{\bar{a}}_{y}^{\prime },{\bar{a}}_{z}^{\prime }\right)$**:**

$$\${\vec{a}}_{x}^{\prime }=\frac{1}{N}\overset{N}{\underset{i=1}{\sum }}\;a_{x}^{{}^{\prime }\left(i\right)}\$$$

$$\${\bar{a}}_{y}^{\prime }=\frac{1}{N}\sum_{i=1}^{N}a_{y}^{{}^{\prime }\left(i\right)}\$$$

$$\${\bar{a}}_{z}^{\prime }=\frac{1}{N}\sum_{i=1}^{N}a_{z}^{{}^{\prime }\left(i\right)}\$$$

For any given case in the validation group, the TLS and reference length L were constructed using only P1, P2, and P3. The preliminary single center anatomical prior was then inversely mapped to the native global RAS system to obtain the predicted coordinates of the center of the Segond fracture bed:

$$\${\hat{P}}_{4}=P_{2}+\left({\vec{a}}_{x}^{\prime }\cdot L\right)e_{x}+\left({\vec{a}}_{y}^{\prime }\cdot L\right)e_{y}+\left({\vec{a}}_{z}^{\prime }\cdot L\right)e_{z}\$$$

In the validation group, the predicted coordinates of the center of the Segond fracture bed were compared with the manually annotated coordinates. The primary evaluation metric was the three-dimensional Euclidean distance error.

### 2.7 Statistical analysis

Multivariate statistical analysis and spatial error quantification methods were employed to characterize the distribution pattern of the fracture bed and to validate the accuracy of the predictive model. Based on the constructed right-handed three-dimensional local coordinate system with origin at the tibial tuberosity, multivariate statistical analysis was performed on the normalized three-dimensional coordinates of the Segond fracture bed center points from the model-development group. The sample multivariate mean vector and three-dimensional covariance matrix were computed to evaluate the spatial dispersion along the X, Y, and Z axes and the linear coupling relationships among the three axes. A 95% three-dimensional confidence ellipsoid was constructed under the assumption of multivariate normality, and the stability and regularity of the standardized three-dimensional spatial distribution of the center of the Segond fracture bed were visually verified in conjunction with three-dimensional point cloud visualization. Additionally, three-dimensional Euclidean distance was employed to quantify the spatial deviation between the model-predicted coordinates and the ground-truth coordinates derived from CT measurements. The mean, maximum, and minimum values of the prediction errors were calculated to perform validation of the localization accuracy of the predictive model, thereby comprehensively evaluating its clinical applicability and spatial localization reliability.

## 3 Results

### 3.1 Standardized three-dimensional distribution of the center of the segond fracture bed

The TLS and N-TLS coordinates of the 15 Segond fracture bed centers in the model-development group are presented in Table 1. After local coordinate transformation and scale normalization, the mean coordinates of the Segond fracture bed centers were (0.8209, 0.4300, −0.0078). This result indicates that, relative to the reference length from P2 to P1, the mean dimensionless positions of the fracture bed center along the X, Y, and Z anatomical axes were approximately 0.8209, 0.4300, and −0.0078, respectively. A 95% three-dimensional confidence ellipsoid was fitted based on the entire normalized feature dataset, with semi-axis lengths of (0.1403, 0.1262, 0.1125) for the three axes. The three-dimensional point cloud visualization (Figure 3) demonstrated that all encoded feature points from the entire sample were concentrated within the 95% confidence ellipsoid surrounding the mean prior center.

**Table 1:** Coordinates of segond fracture bed centroid in the tibial local coordinate system (TLS) and the normalized tibial local coordinate system (N-TLS) (modeling cohort, *n* = 15).

No.	TLS (X, Y, Z, mm)	Reference length L (mm)	N-TLS (X, Y, Z)
1	(35.5862, 19.2541, −7.4678)	42.4889	(0.8375, 0.4532, −0.1758)
2	(40.7989, 26.8808, −1.9758)	50.8914	(0.8017, 0.5282, −0.0388)
3	(47.0449, 16.0104, −8.0220)	54.9893	(0.8555, 0.2912, −0.1459)
4	(26.8285, 25.2052, −4.9348)	43.0445	(0.6233, 0.5856, −0.1146)
5	(42.4661, 25.9505, 5.8735)	41.8715	(1.0142, 0.6197, 0.1402)
6	(46.6220, 22.8013, 11.6857)	55.5742	(0.8389, 0.4103, 0.2103)
7	(30.5971, 42.3924,8.2466)	47.8907	(0.6389, 0.8852, 0.1722)
8	(44.7740, 12.8095, −7.3289)	47.3331	(0.9459, 0.2706, −0.1548)
9	(48.2095, 18.9998, −3.4194)	49.8554	(0.9670, 0.3811, −0.0686)
10	(49.5214, 16.5210, −10.7054)	55.8415	(0.8868, 0.2959, −0.1917)
11	(48.0657, 16.6593, −5.6257)	51.3620	(0.9358, 0.3243, −0.1095)
12	(31.6947, 11.8351, 4.9797)	43.5056	(0.7285, 0.2720, 0.1145)
13	(49.2776, 24.1854, 5.1242)	56.5868	(0.8708, 0.4274, 0.0906)
14	(43.1132, 22.9041, 8.2588)	54.8201	(0.7864, 0.4178, 0.1507)
15	(36.3375, 18.0265, 0.1571)	62.3906	(0.5824, 0.2889, 0.0025)

**Figure 3:**
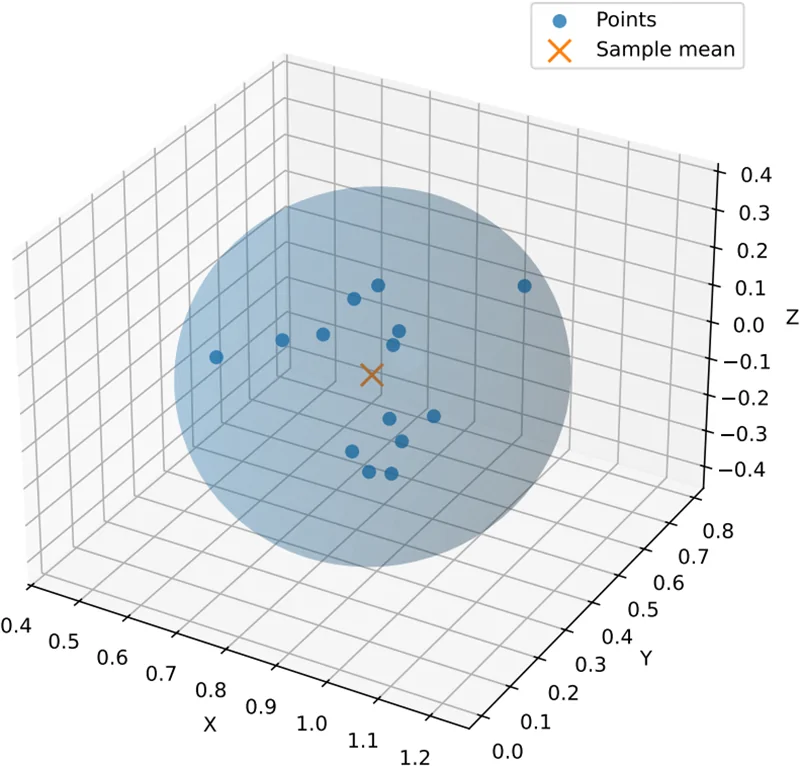
Three-dimensional point cloud visualization of the segond fracture features after local coordinate transformation and scale normalization.

### 3.2 Localization error in the validation group

After inputting the coordinates of P1, P2, and P3 from the validation group cases into the deterministic geometric estimator, the corresponding predicted coordinates of P4 were obtained Table 2. Compared with the CT-annotated coordinates, the model achieved a mean prediction error of 3.21 mm, with a standard deviation of 1.23 mm and a median error of 2.75 mm. The maximum prediction error was 5.33 mm, and the minimum was 1.68 mm. All 15 validation samples had prediction errors below 6.0 mm, with 13 cases (86.7%) exhibiting errors less than 4.0 mm.

**Table 2:** Statistical results of 3D Euclidean distances between predicted and ground-truth segond fracture centroid coordinates of the validation cohort under the native global RAS coordinate system (*n* = 15).

No.	Ground-truth centroid (X, Y, Z, mm)	Predicted centroid (X, Y, Z, mm)	Euclidean error (mm)
1	(145.31, 53.51, −34.4089)	(144.31, 52.11,−35.78)	2.20
2	(109.77, −472.17, 663.09)	(106.69,−475.25,660.01)	5.33
3	(129.93, −463.18, 940.5867)	(128.93,−464.68,938.51)	2.75
4	(128.62, −477.64, 613.62)	(127.62, −478.84,612.35)	2.01
5	(−137.10, −289.96, 921.5201)	(−137.60,−290.76,920.13)	1.68
6	(−106.25, −702.73, 973.6971)	(−107.25,−704.53,972.21)	2.54
7	(−114.74, 153.21, −144.5847)	(−115.74,151.21,−147.62)	3.77
8	(132.36, −460.37, 570.96)	(131.36,−461.87,569.44)	2.36
9	(84.51, 680.28, −1388.36)	(85.71,682.08,−1390.48)	3.03
10	(205.74, −288.50, 676.16)	(206.74 −286.9 674.54)	2.49
11	(37.16, −38.01, 691.47)	(39.01,−40.61, 694.68)	4.53
12	(−126.53, −56.58, 939.95)	(−125.53,−54.88,937.95)	2.81
13	(−188.73, −42.92, 718.01)	(−191.23,−45.92,715.07)	4.89
14	(−170.67, −26.69, 735.01)	(−173.67,−30.19,732.48)	5.26
15	(56.59, −79.73, 687.35)	(57.59,−78.23,685.69)	2.45

## 4 Discussion

In this study, a standardized three-dimensional CT anatomical geometric encoding framework was constructed based on superficial tibial osseous landmarks, with the core objective of achieving a generalizable three-dimensional quantitative representation of the Segond fracture bed target. The entire geometric transformation pipeline can be encapsulated as an automated inference subroutine, well-suited for integration into knee AI-assisted surgical systems. The core innovation of this framework lies in the selection of three superficial bony prominences that are precisely palpable intraoperatively as unified anatomical references, offering substantial advantages for clinical translation. After completion of three-point intraoperative identification and registration, the knowledge-driven geometric inference module developed in this study can automatically output the three-dimensional coordinates of the Segond fracture bed, providing standardized quantitative targets for anatomical reconstruction of the anterolateral ligament (ALL) and precise reduction and internal fixation of Segond fracture fragments.

Research on the localization and measurement of the Segond fracture bed spans several decades. Claes et al., through cadaveric dissections, suggested that the femoral attachment of the ALL is located slightly anterior to the lateral collateral ligament attachment (10). Daggett et al. found that the femoral attachment of the ALL lies posteroproximal to the lateral femoral epicondyle (11). However, these studies employed one-dimensional linear distance scalars to describe anatomical positions without correcting for the systematic measurement bias introduced by the natural curvature of the tibial plateau. Moreover, such metrics cannot be rapidly deduced intraoperatively using superficial osseous landmarks. Helito et al. utilized radiographic imaging to localize the Segond fracture bed, using the distance from the fracture bed to the anterior and posterior margins of the tibial plateau on lateral radiographs (12). In our previous study, we achieved two dimensional localization of the Segond fracture bed based on the tibial tuberosity and Gerdy tubercle (13). Nevertheless, two-dimensional radiographic measurement has inherent geometric limitations: the anterior-posterior extreme points of the tibial plateau are non-coplanar under X-ray projection. Projected distances exhibit systematic distortion relative to true three-dimensional spatial lengths. Therefore, two-dimensional methods cannot accurately characterize the actual three-dimensional spatial position of the Segond fracture bed. They fail to provide reliable input features for surgical intelligent-planning systems and cannot precisely guide AI-driven automatic planning of the tibial tunnel for the anterolateral ligament (ALL).

Precise localization of the characteristic anatomical region of the anterolateral proximal tibia associated with Segond fracture holds critical value for AI-assisted prediction of anterolateral complex injuries of the knee and automated generation of personalized surgical plans (14). Fares et al. developed a novel computational method to customize synthetic bone plates by integrating CT imaging and computational modeling. Their approach addresses challenges related to anatomical variability and precision in orthopedic surgery, improving surgical efficiency, accuracy, and clinical outcomes (15). We adopted a similar strategy to resolve the positional accuracy issues for Segond fracture assessment. Segond fracture serves as a characteristic imaging marker of ALL tibial avulsion; however, ALL substance injuries may occur without bony avulsion, and some patients with combined ACL and severe ALL injury present only with a positive pivot-shift test without radiographic evidence of Segond fracture (16–18). The Segond fracture bed corresponds to the native ALL tibial attachment region, and the fracture plays a significant role in guiding ALL reconstruction site selection (19, 20). Malpositioning of the reconstruction site can continuously alter the ligament tension curve throughout knee range of motion, substantially increasing the risk of postoperative graft laxity, rupture, and other complications (21, 22). The standardized geometric encoding and inference framework established in this study requires only the input of three superficial osseous landmark coordinates to complete automated three-dimensional feature encoding of the ALL tibial attachment. Leveraging the preliminary single center anatomical prior derived from the model-development group, the framework can automatically infer a virtual Segond target zone in patients without avulsion fracture, thereby providing AI-recognizable standardized three-dimensional anatomical features for ALL anatomical reconstruction in cases without bony fragmentation.

In the field of three-dimensional anatomical quantification of the proximal tibia, traditional measurement systems typically establish orthogonal reference planes using the tibial mechanical axis as the vertical benchmark (23). Such coordinate systems heavily depend on the intact tibial articular surface and diaphyseal anatomy, rendering them difficult to implement intraoperatively: in patients with obesity or traumatic hemarthrosis, the boundaries of the tibial plateau cannot be clearly palpated, precluding the real-time osseous registration required for intelligent navigation systems. This study innovatively selected three readily identifiable and superficially palpable osseous landmarks—the midpoint of the lateral margin of the tibial tuberosity, the most lateral prominence of the fibular head, and the highest point of Gerdy's tubercle—to construct a stable anatomical reference plane and complete three-dimensional geometric feature encoding of the Segond fracture bed. These three osseous landmarks are compatible with both intraoperative manual palpation registration and unambiguous automated annotation on CT three-dimensional reconstruction images, serving as a versatile anatomical interface connecting clinical surgery and AI-based imaging analysis platforms. Concurrently, a scale normalization preprocessing module was introduced, using the reference segment length from the tibial tuberosity to the fibular head to perform dimensionless scaling of features, thereby completely eliminating feature scale bias arising from inter-individual differences in skeletal dimensions and body habitus, and ensuring that data from different patients can be uniformly fed into the AI inference pipeline. Once the three osseous landmarks are registered intraoperatively, the inference program can output the predicted coordinates of the Segond fracture bed in real time, assisting surgeons in precise preparation of the ALL tibial tunnel position. In the long term, the entire standardized deterministic geometric estimator can be fully integrated into intraoperative robotic navigation systems: after the surgeon performs simple three-point registration, the system can automatically deduce the virtual ALL attachment zone in patients without fracture, enabling real-time intraoperative AI-assisted target zone localization.

The above accuracy metrics verify that this knowledge-driven geometric inference prediction model achieves a mean localization error slightly lower than the 3.5-millimeter interobserver variability threshold reported in prior literature (24). Nevertheless, this study has certain limitations. First, the model-development subset consisted of only 15 patients and the held-out validation subset included another 15 patients. The limited sample size precludes establishing a generalizable preliminary single center anatomical prior or deriving clinically validated accuracy thresholds; therefore, the findings cannot be directly implemented for large scale clinical application at the present stage. Second, only patients with left knee Segond fractures were enrolled in this study. The established modeling and validation results cannot be extrapolated to patients with Segond fractures of the right knee. This work is primarily a methodological study that demonstrates the feasibility of the proposed approach as well as favorable positional-error performance. It lays a methodological foundation for future large sample size investigations and AI enabled surgical navigation.

In summary, this study established a standardized three-dimensional CT anatomical coordinate framework based on three tibial osseous landmarks and developed a deterministic deterministic geometric estimator for the center of the Segond fracture bed using the preliminary single center anatomical prior derived from the model-development group. The normalized three-dimensional anatomical features generated by this framework can serve as a unified quantitative input for Segond fracture and ALL attachment localization, providing a standardized and reusable computational reference for intelligent preoperative planning of anterolateral knee injuries and automated intraoperative target zone delineation in AI-assisted navigation.

## 5 Conclusion

In this study, a standardized three dimensional CT anatomical coordinate framework was constructed based on three tibial osseous landmarks, and a deterministic deterministic geometric estimator for the center of the Segond fracture bed was established using the preliminary single center anatomical prior derived from the model development group. In the independent validation group, the model achieved a mean three dimensional localization error of 3.21 mm. This method is characterized by its transparent computational process, interpretable parameters, and ease of algorithmic implementation, thereby serving as a candidate foundational tool for preoperative planning in patients with high grade knee pivot shift, computer assisted surgical navigation, and anatomical registration in future digital knee models.

## Data Availability

The raw data supporting the conclusions of this article will be made available by the authors, without undue reservation.
